# Chronic Granulomatous Herpes Encephalitis in a Child with Clinically Intractable Epilepsy

**DOI:** 10.1155/2012/849812

**Published:** 2012-08-07

**Authors:** James R. Hackney, D. Keith Harrison, Curtis Rozzelle, Suthida Kankirawatana, Pongkiat Kankirawatana, Cheryl Ann Palmer

**Affiliations:** ^1^Division of Neuropathology, Department of Pathology, University of Alabama at Birmingham, 1960 6th Avenue South, PD6A 175E, Birmingham, AL 35294, USA; ^2^Department of Pathology, Children's Hospital of Alabama, Birmingham, AL 35233, USA; ^3^Division of Neurosurgery, Department of Surgery, University of Alabama at Birmingham, Birmingham, AL 35294, USA; ^4^Division of Pediatric Allergy, Immunology, Rheumatology and Pulmonary Medicine, Children's Hospital of Alabama, Birmingham, AL 35233, USA; ^5^Department of Neurology, Children's Hospital of Alabama, Birmingham, AL 35233, USA

## Abstract

Most patients with herpes simplex virus Type I encephalitis experience an acute, monophasic illness. Chronic encephalitis is much less common, and few late relapses are associated with intractable seizure disorders. A 10-year-old boy was admitted to our institution for intractable epilepsy as part of an evaluation for epilepsy surgery. His history was significant for herpes meningitis at age 4 months. At that time, he presented to an outside hospital with fever for three days, with acyclovir treatment beginning on day 4 of his 40-day hospital course. He later developed infantile spasms and ultimately a mixed seizure disorder. Video electroencephalogram showed a Lennox-Gastaut-type pattern with frequent right frontotemporal spikes. Imaging studies showed an abnormality in the right frontal operculum. Based on these findings, he underwent a right frontal lobectomy. Neuropathology demonstrated chronic granulomatous inflammation with focal necrosis and mineralizations. Scattered lymphocytes, microglial nodules and nonnecrotizing granulomas were present with multinucleated giant cells. Immunohistochemistry for herpes simplex virus showed focal immunoreactivity. After undergoing acyclovir therapy, he returned to baseline with decreased seizure frequency. This rare form of herpes encephalitis has only been reported in children, but the initial presentation of meningitis and the approximate 10-year-time interval in this case are unusual.

## 1. Introduction

Herpes simplex virus (HSV) Type I is an important cause of encephalitis in both adults and children outside of the neonatal period [1, 2]. While most patients experience an acute, monophasic disease course, recurrent/relapsing forms of acute encephalitis as well as chronic encephalitis with development of intractable epilepsy have been described [3, 4]. Some of these are late relapses that have been associated with intractable seizure disorders requiring surgical intervention. Prior reports of such patients describe neuropathological findings ranging from astrogliosis, microgliosis, perivascular lymphocytic infiltrates, and necrosis similar to acute HSV infection to a more chronic granulomatous encephalitis with mineralization [2, 4, 5]. We report the case of a 10-year-old boy with intractable epilepsy and a remote history of acute herpes meningitis at age 4 months. Surgical resection of a right frontal lobe seizure focus demonstrated chronic granulomatous encephalitis with positive immunohistochemical results for HSV. 

## 2. Patient Presentation

**Figure 1:**
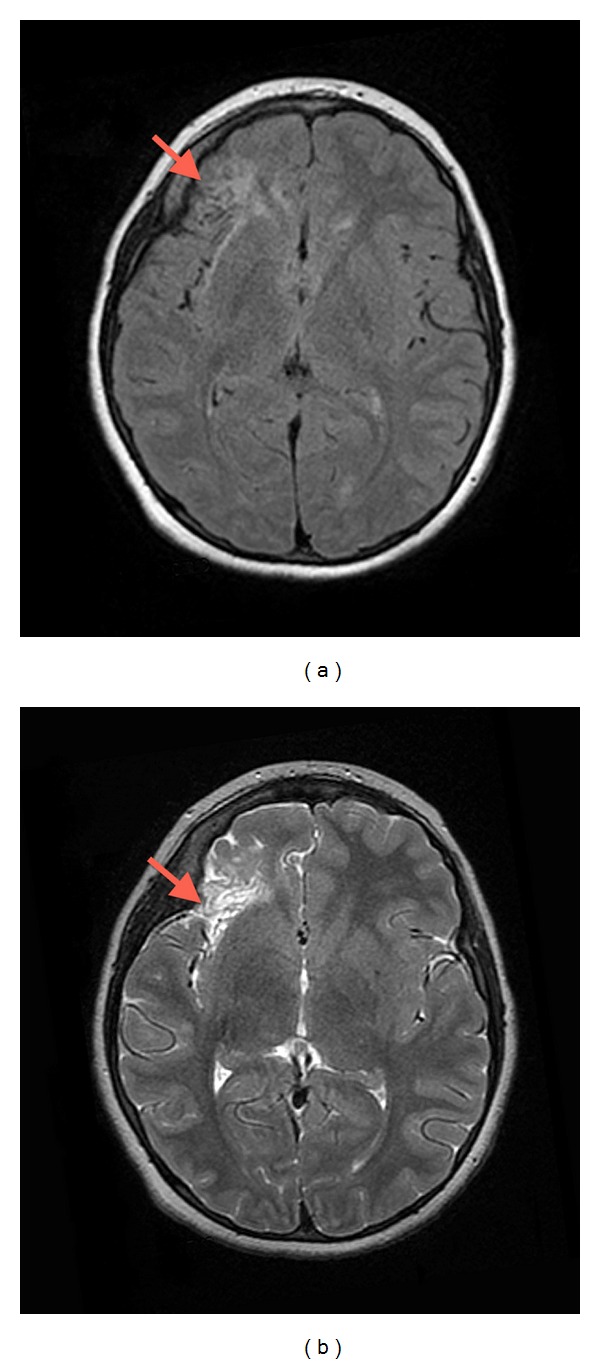
Magnetic resonance imaging studies reveal an abnormality in the right frontal operculum [(a) Axial T2 FRFSE; (b) Axial T2 FLAIR].

A 10-year-old boy was admitted to The Children's Hospital of Alabama for medically intractable epilepsy as part of a presurgical evaluation for epilepsy surgery. His medical history was significant for herpes meningitis at age 4 months. At that time, he presented with fever for 3 days, with acyclovir treatment beginning on day 4 of his 40-day hospital course. There was no known family history of immunodeficiency, predisposition to viral infections, or epilepsy. While his developmental status had been normal at birth, he later experienced profound developmental delay beginning at about 6 months of age. He could walk but preferred to crawl, and his attention was poor. He had no language but was able to verbalize his needs in a manner interpretable by his mother. He lived at home and attended special education classes. He subsequently developed infantile spasms and ultimately a mixed seizure disorder characterized by absent, blank stares one to three times daily, sudden drop attacks about twice per month, and tonic/clonic seizures once or twice weekly. Recent video electroencephalogram showed a Lennox-Gastaut-type pattern with focal features of frequent runs of right frontotemporal spikes. His captured clinical seizure semiology demonstrated 2 types of seizures: dialeptic seizures and left arm fencing. Clinically, his seizure types also included frequent drop attacks with multiple episodes of status epilepticus. He had experienced several breakthroughs despite several drug changes and a current regimen of six antiepileptic medications. His regimen included levetiracetam 750 mg twice daily, lacosamide 200 mg twice daily, topiramate 100 mg twice daily, zonisamide 300 mg at bedtime, lorazepam 1 mg as needed, and rectal diazepam 12.5 mg as needed. In addition, a ketogenic diet had been tried without appreciable benefit. His MRI scan displayed an abnormality in the anterior portion of the right insula and right frontal operculum (Figure 1). The cortex in this area was abnormally thin with increased T2 signal and a ribbon of low T2 signal around the periphery. There was some deficiency of subfrontal white matter in this region, but the abnormal area was fairly sharply demarcated. FLAIR images demonstrated some increased signal in the adjacent white matter. There was no evidence of hemorrhage or infarct. Based on these findings, the patient underwent a right frontal craniotomy for a partial frontal lobectomy. Recovery was complicated by a cerebrospinal fluid (CSF) leak requiring reoperation 1.5 weeks postsurgery. Upon reexploring the craniotomy, the previously normal-appearing dura had large, spontaneous fenestrations remote from the suture line. Postoperative herpes viral cultures and CSF herpes polymerase chain reaction (PCR) studies were negative. CSF and serum herpes antibody studies were not performed. Because of the postsurgical neuropathological findings, the patient also underwent a full, 21-day course of intravenous acyclovir therapy. This consisted of a 21-day course of intravenous acyclovir that began at 20 mg/kg but was reduced to 10 mg/kg after the surgical procedure to repair the CSF leak. This was followed by a return to neurological baseline and the patient was rendered seizure-free for approximately one month. However, this was followed by a return of seizure activity, up to several per day, in spite of the administration of multiple seizure medications. Immunological workup demonstrated a normal distribution and numbers of T, B, and NK lymphocytes, and normal functional studies for T-cell subsets and NK cells. Toll-like receptor (TLR) functional testing was normal for TLR-1–TLR-8, including TLR-3, abnormalities of which have been associated with HSV-1 susceptibility. He is now one year postsurgery with no change in his preoperative electroencephalogram. He is being considered for placement of a vagus nerve stimulator.

## 3. Neuropathology

**Figure 2:**
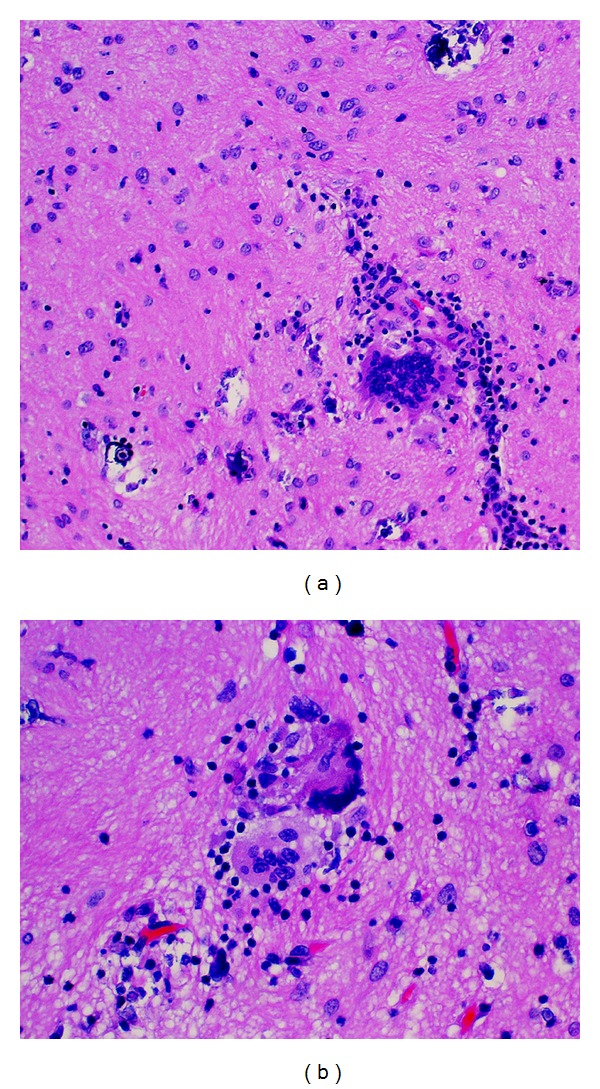
Histopathologic sections reveal chronic inflammation, astrogliosis, and scattered mineralizations [(a) H&E ×200]. Frequent multinucleated giant cells were present [(b): H&E ×400].

**Figure 3:**
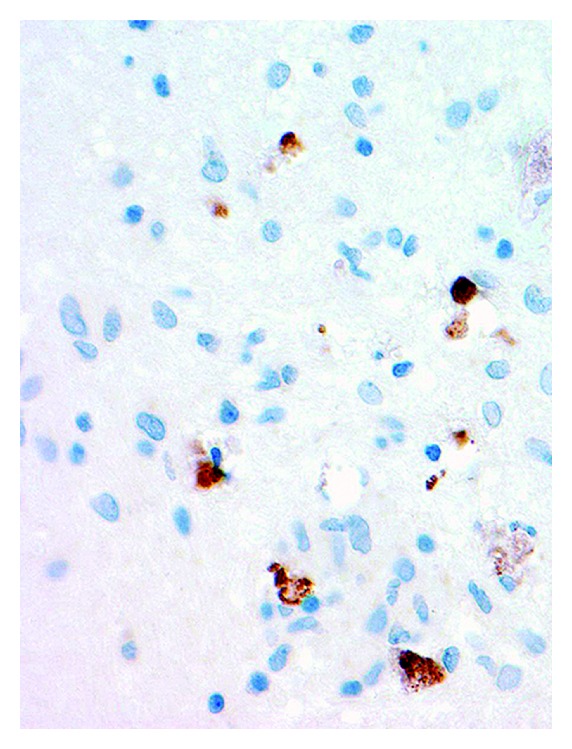
Immunohistochemistry for herpes simplex virus I/II is positive in scattered cells [HSV ×200].

Neuropathological examination of the right frontal lesion demonstrated chronic granulomatous inflammation with foci of necrosis and mineralization. Scattered clusters of lymphocytes, microglial nodules, and small, discrete nonnecrotizing granulomas were present with occasional multinucleated giant cells (Figure 2). Some giant cells had mineralized material within the cytoplasm. These changes were set within both gliotic cerebral cortex and superficial white matter. Immunohistochemical staining for herpes simplex virus (HSV I/II, Cell Marque, Rocklin, California) demonstrated focal nuclear and cytoplasmic immunoreactivity within scattered neuronal and glial cells (Figure 3). The specificity of this tissue marker is such that cross reactivity may be seen with varicella-zoster virus at high concentrations, but cross-reactivity with cytomegalovirus or Epstein-Barr virus is not seen. Special staining for fungi and immunohistochemical staining for cytomegalovirus were also performed and both were negative.

## 4. Discussion

Late relapses of herpes encephalitis are uncommon and are usually characterized by fever, drowsiness, electroencephalogram changes, and increased seizure activity, with or without CSF evidence of HSV presence [3, 5]. Both postinfectious immunoinflammatory disease and viral reactivation have been suggested as potential pathogenetic mechanisms [5]. Evidence cited as indicative of virus reactivation includes detection of HSV in CSF or tissue using immunohistochemistry or PCR, and radiographic or neuropathologic evidence of new typical herpetic cortical lesions [5]. In cases of presumed postinfectious immunoinflammatory disease, findings include diffuse white matter involvement and cerebral edema with absence of the above features of viral reactivation. In the pediatric age group, this mechanism of late relapse has been confirmed in only one case, a 17-year-old boy who relapsed 8 months after primary treatment [5]. 

Spiegel et al. [5] reported one case of late relapse of herpes simplex viral encephalitis and reviewed an additional 12 cases found in the literature from the previous 15 years. They concluded that reactivation of latent virus was the most likely cause of late relapse, based on radiologic and neuropathological autopsy findings. Lellouch-Tubiana et al. [2] had earlier reported 3 patients in France who were not included in the review of Spiegel et al. [5]. These patients demonstrated late relapse 3–10 years postinfection. Two of these three patients had evidence of viral recrudescence by CSF viral antibody titers, but neuropathological demonstration of virus in tissue by immunohistochemistry or PCR analysis was not performed. The brain of the third patient demonstrated atrophy and white matter changes that correlated clinically with worsening epilepsy more consistent with postinfectious immunoinflammatory disease. Adamo et al. [7] recently reported a late relapse after a 13-year latency, confirmed by biopsy and positive HSV PCR results on frozen biopsy tissue, although CSF PCR studies and tissue immunohistochemistry had been negative for HSV. Cases of histologically verified chronic granulomatous herpes simplex encephalitis are summarized in Table 1.

The identification of HSV by immunoperoxidase and the findings at reoperation in our patient support the interpretation of viral reactivation as the cause of our patient's deterioration rather than residual damage from the initial insult. Negative culture results from our patient most likely represent the general difficulty in culturing virus from tissue specimens combined with the likelihood of a low viral load in both tissue and CSF. Our patient's return to baseline following antiviral therapy may also argue in favor of reactivation, although the clinical course was certainly affected by the epilepsy surgery. An additional point of interest is that one of the patients discussed by Spiegel et al. [5] had a latency period of 8.5 years, while the patient presented by Adamo et al. [7] experienced a latency period of over 13 years, the longest reported to date with neuropathological confirmation. Our case, with a latency period of 10 years, joins these cases in illustrating the extended latency period that can rarely be seen with herpes virus reactivation.

## 5. Conclusions

Chronic granulomatous herpes encephalitis is a rare form of herpes encephalitis seen only in children, usually as a late complication or relapse characterized by intractable seizures, neurological deterioration, and new neuroimaging findings. Neuropathological examination demonstrates features consistent with viral reactivation, including virus identifiable within neurons by immunohistochemistry or PCR analysis. Patients benefit from retreatment with acyclovir, with or without surgery for control of intractable epilepsy. Such treatment dramatically reduced our patient's seizure activity and returned him to his neurological baseline. His presentation 10 years after the initial episode is unusual, being the longest yet reported latency period with neuropathological confirmation by immunohistochemistry.

## Figures and Tables

**Table 1:** Histologically verified cases of chronic granulomatous herpes simplex encephalitis.

Source	Latency	Presentation	Treatment	Course
Jay et al. [6] (Pt. 2)	2.5 years	Intractable Seizures	Temporal lobectomy	Not available
Love et al. [4] (Pt. 1)	4.5 years	Intractable Seizures	Hemispherectomy	Marked Improvement
Love et al. [4] (Pt. 2)	10 years	Intractable Seizures	Temporal lobe excision × 2 + Acyclovir	Marked Improvement
Love et al. [4] (Pt. 3)	2 months	Congenital HSV2 Skin infection^∗^	Topical Acyclovir	Sudden death
Adamo et al. [7]	14 years	Intractable Seizures	Biopsy + Acyclovir	Marked Improvement
Current case	10 years	Intractable Seizures	Frontal lobectomy + Acyclovir	Minimal improvement

^∗^All cases had documentation of HSV1 infection except Patient 3 from Love et al. [4] who had congenital HSV2 infection eventuating in sudden death, with granulomatous herpes encephalitis diagnosed at autopsy.
